# Genomic and Personalized Medicine Approaches for Substance Use Disorders (SUDs) Looking at Genome-Wide Association Studies

**DOI:** 10.3390/biomedicines9121799

**Published:** 2021-11-30

**Authors:** Danilo Cozzoli, Alessia Daponte, Salvatore De Fazio, Vincenza Ariano, Maria Rita Quaranta, Vincenzo Leone, Angelo Ostuni, Margherita Casanova, Claudia Rita Catacchio, Mario Ventura, Francesco Montinaro

**Affiliations:** 1 Associazione Comunità Emmanuel ETS, Via don Bosco 16, 73100 Lecce, Italy; danilo.cozzoli@gmail.com (D.C.); leonevincenzo@emmanuel.it (V.L.); 2 Dipartimento di Biologia, Università degli Studi di Bari, Via Orabona 4, 70124 Bari, Italy; a.daponte1@studenti.uniba.it (A.D.); claudiarita.catacchio@uniba.it (C.R.C.); 3 Struttura Sovradistrettuale Dipendenze Patologiche ASL Brindisi, Via Santa Teresa, 72100 Brindisi, Italy; salvdefazio@yahoo.it; 4 Dipartimento Dipendenze Patologiche, ASL Taranto, Via Ancona, 70100 Taranto, Italy; cinziariano@homail.it; 5 SERD Martina Franca, ASL Taranto, Via Orazio Flacco 127, 74015 Martina Franca, Italy; mariaritaquaranta@yahoo.it; 6 U.O. Medicina Trasfusionale AOU Policlinico Bari, Piazza Giulio Cesare, 70125 Bari, Italy; angelostuni@gmail.com (A.O.); margheritacasanova@virgilio.it (M.C.); 7 Institute of Genomics, University of Tartu, 51010 Tartu, Estonia

**Keywords:** drug addiction, pharmacogenomics, genetics

## Abstract

Drug addiction, or substance use disorder (SUD), is a chronic, relapsing disorder in which compulsive drug-seeking and drug-taking behaviour persist despite serious negative consequences. Drug abuse represents a problem that deserves great attention from a social point of view, and focuses on the importance of genetic studies to help in understanding the genetic basis of addiction and its medical treatment. Despite the complexity of drug addiction disorders, and the high number of environmental variables playing a role in the onset, recurrence, and duration of the symptoms, several studies have highlighted the non-negligible role of genetics, as demonstrated by heritability and genome-wide association studies. A correlation between the relative risk of addiction to specific substances and heritability has been recently observed, suggesting that neurobiological mechanisms may be, at least in part, inherited. All these observations point towards a scenario where the core neurobiological factors of addiction, involving the reward system, impulsivity, compulsivity, stress, and anxiety response, are transmitted, and therefore, genes and mutations underlying their variation might be detected. In the last few years, the development of new and more efficient sequencing technologies has paved the way for large-scale studies in searching for genetic and epigenetic factors affecting drug addiction disorders and their treatments. These studies have been crucial to pinpoint single nucleotide polymorphisms (SNPs) in genes that affect the reaction to medical treatments. This is critically important to identify pharmacogenomic approaches for substance use disorder, such as *OPRM1* SNPs and methadone required doses for maintenance treatment (MMT). Nevertheless, despite the promising results obtained by genome-wide association and pharmacogenomic studies, specific studies related to population genetics diversity are lacking, undermining the overall applicability of the preliminary findings, and thus potentially affecting the portability and the accuracy of the genetic studies. In this review, focusing on cannabis, cocaine and heroin use, we report the state-of-the-art genomics and pharmacogenomics of SUDs, and the possible future perspectives related to medical treatment response in people that ask for assistance in solving drug-related problems.

## 1. Introduction

Several decades of twin, adoption and family studies converge on the fact that part of the differences in behavioural traits such as depression, personality and cognitive abilities, have a genetic basis, in a complex interaction between genetics and environmental variables [1]. Although the significance and impact of the heritability estimates are still matters of intense evaluation and debate, the role of genetics in many behavioural traits has been thoroughly investigated using genotype arrays or genome sequences, in genome-wide association studies (GWAS) [2]. In GWAS, differences in the allele frequency of genetic variants, copy-number variants or sequence variation, or correlation between allele frequency and quantitative traits are tested. Despite the enormous effort, the high polygenicity characterizing many behavioural traits has prevented a reliable and replicable identification of the genetic variants underlying the variability of such traits. In addition, even when promising results were obtained, the variation explained was only a small fraction of the total inferred in twin studies, a problem often referred to as “missing heritability” [3,4,5,6,7]. These discrepancies in heritability estimates may have many different causes, such as the role of rare variants or the lack of statistical power provided by association studies, mostly due to a relatively low number of tested cases. Nevertheless, the development of GWAS with tens or hundreds of thousands of tested individuals, together with the development of risk scores including not only the statistically significant hits, are finally giving very promising results [8]. As an example, a recent study aiming to identify the genetic SNPs having a role in schizophrenia spectrum disorder identified almost 200 variants that, taken together, explain 25% of the total variability [9]. In this perspective, it may be possible that future studies characterized by large sample size, together with the refinement of statistical algorithms and the analysis of whole genome sequencing data are expected to improve the applicability of these results.

**Figure 1:**
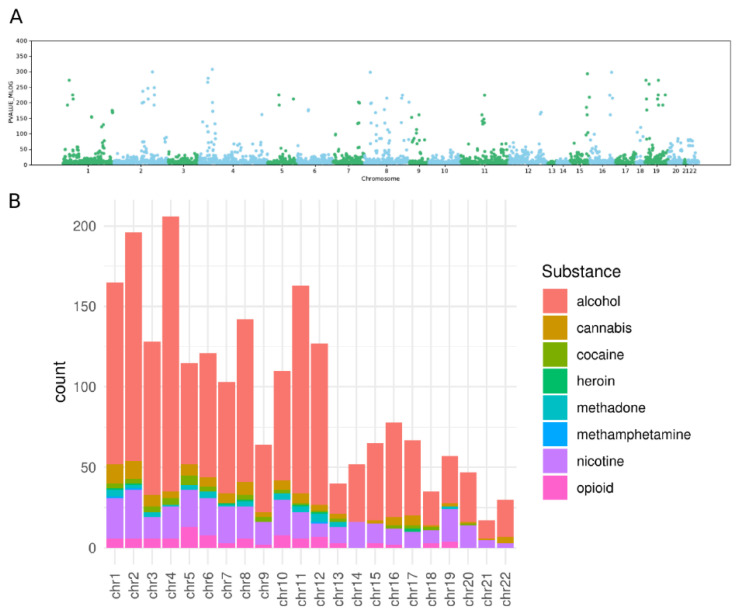
(**A**). Manhattan plot of the SNPs associated with substances in relation to the opposite of the logarithm of their *p*-value (PVALUE_MLOG). From the GWAS catalog, for autosomes, SNPs with the following keywords in the column “reported trait” were extracted: opioid, alcohol, cannabis, cocaine, heroin, methadone, methamphetamine, nicotine and tobacco. We removed associations with missing reported genomic location. (**B**). Histogram of the count of SNPs associated with specific substances (see colour code) per chromosome. For each chromosome the number of associated markers has been stratified for each substance.

Among the many behavioral traits of clinical relevance, those related to substance use, abuse or addiction have been the subject of intense investigation given their high medical and social impact. Traits related to smoking and drinking behaviours were the most investigated, and led to the identification of many SNPs related to alcohol (ADH family) or nicotine metabolism, putting the basis for a better exploration of the physiological dynamics of addiction [10,11]. On the other hand, the advancement of the study of possible genetic variants linked to other substance diseases has been delayed and challenged by many different limitations and challenges, such as the high level of stigmatization for individuals affected by drug disorders, the complexity of the traits under investigation, together with all the limitations affecting the interpretation of heritability and GWAS. A preliminary analysis of the genetic markers available in the GWAS catalog presented in 2021 associated with any traits containing the word alcohol, nicotine or tobacco, cocaine, cannabis, heroin, opioid, methadone, and methamphetamine revealed that out of 3927 associations, only 331 were related to substances other than alcohol and nicotine or tobacco (Figure 1).

Only recently, thanks to the creation of multicentre consortia and biobanks recruiting a considerable number of individuals with SUDs, such studies were made possible, raising the hope that soon, genetic discoveries may contribute, at least in part, to the prevention of substance use disorders. Furthermore, the understanding of the genetic architecture underlying the variability of response to different treatments for substance use may help to develop a genetic-informed therapeutic strategy and to better characterize how the environmental variables are related to this behaviour.

In this review, focusing on GWAS for cannabis, cocaine and heroin uses, we describe the possible future perspectives related to addiction medical treatment response in people that ask for assistance in solving drug-related problems.

## 2. The Genetics of Cannabis Use

Cannabis is the most cultivated, consumed, and trafficked substance with psychoactive properties, with roughly 200 million people using cannabis in 2019, representing 4% of the global population [12]. In the last decades, despite its general negative effects, both acute and chronic, cannabis has become legal or decriminalised in many countries.

From a genetic perspective, family-based studies have inferred a heritability h^2^ between 51% and 71% [13,14], highlighting the non-negligible role of genetic variation on cannabis use disorders. However, despite being widely consumed, GWAS investigating cannabis use and characterised by robust methodology and large sample size were developed only recently.

A study focusing on the lifetime prevalence and analysing 184,765 individuals, identified eight markers, belonging to seven genes (*CADM2*, *SDK1*, *ZNF704*, *NCAM1*, *RABEP2*, *ATP2A1*, *SMG6*) significantly associated (*p* < 5 × 10^−8^) with lifetime cannabis use. Furthermore, the analysis of single gene associations and gene expression levels extended the list of associated genes to 45. Among these, the most striking signal is in *CADM2*, located on chromosome 3, and highly expressed in different areas of the brain. In detail, the gene is part of the immunoglobulin (Ig) superfamily and encodes for a member of the synaptic cell adhesion molecule 1 (SynCAM). It has been found to be associated with several behavioural traits (GWAS catalog [15,16]), such as alcohol consumption [17], smoking status and initiation, and drug use [18]. Another strong candidate that emerged from the study was NCAM1, located in the *NCAM1–TTC12–ANKK1–DRD2* gene cluster, which has been previously linked to smoking and alcohol use [19], but also to psychiatric disorders [20,21]. Although these genes may play a role in the exposure to cannabis, it is not clear whether and how they are relevant in drug use disorders, such as abuse and addiction. Similarly, a genome-wide analysis of ~25,000 individuals from nine different cohorts identified five SNPs in the Calcium-transporting ATPase gene (ATP2C2) associated to the age at first use, suggesting that calcium signalling mechanism may have a role in substance use disorders [22]. The observation that a similar study on 6744 individuals did not identify any significantly associated SNP highlighted the importance of the sample size when analysing a highly polygenic trait [23]. In this context, GWASs aiming at the investigation of genetic traits underlying cannabis dependence characterized by a relatively high sample size were developed only recently. In a survey comparing cannabis-dependent and cannabis-exposed individuals of European descent from five cohorts, several SNPs in chromosome 10 were found to be significantly associated with cannabis dependency. The associated SNPs are mapped to a regulatory domain including 12 genes, with a consistent proportion of them being expressed in the brain. In addition, one of the significantly associated SNPs (rs1409568) is located within an active enhancer [24]. However, these findings were not replicated in a different sample of European individuals, and only weakly replicated in African American individuals [24]. More importantly, this study did not find any overlap with previously reported associated variants. On the contrary, a GWAS of cannabis use disorders in 2387 cases and 48,985 controls and replicated in a cohort of Icelandic individuals found associated variants in the gene cholinergic receptor nicotinic α2 subunit (CHRNA2) [25]. Given that there are no reported links between the nAChR α2 subunit and cannabis consumption, it might be possible that some substance contained in cannabis can interact with the subunit. Alternatively, it could be possible that cannabis can indirectly affect the subunit, through the mediation of an endogenous ligand, such as acetylcholine. Lastly, it is possible that CHRNA2 and cannabinoid receptor genes are biologically linked, as suggested by a strong negative correlation between the expression of CHRNA2 and CNR1 in six human brains [25].

However, although genotyping arrays demonstrated their pivotal role in translation medicine, they prevent the identification of rare associated markers which under highly polygenic models [26,27] could contribute to most of the variance of the considered trait. In this context, an early study analysing ~400 cases (200 individuals per cohort) and ~2000 controls identified two genomic regions associated with cannabis dependence, highlighting an enrichment for regions within or near genes playing a role in cell adhesion or potassium channel activity [28] (Table 1).

In conclusion, despite some promising insights of GWAS investigating the genetic mechanism of cannabis related traits; the small sample size, the inhomogeneity in methodology and the lack of whole genome analysis have limited the full understanding of the genetic role and biological dynamics of the trait. In addition, future studies integrating genomic data, gene expression analysis in different tissues, methylation patterns, and metabolome data are expected to shed light to this complex phenomenon.

## 3. The Genetics of Cocaine Use

Given its severe social and medical impact, the investigation of the genetic traits underlying cocaine use have received increasing attention. In this perspective, the development of a long-term collaborative consortium committed to evaluating addiction, genetics and environment has significantly helped in the advancement of the subject [29]. The first genomic investigations started in the early 2000s, using a limited number of markers genotyped in small nucleus families. Despite their limited power, these genome wide linkage studies identified a few suggestive loci associated with cocaine dependence (CD) and linked traits (e.g., cocaine-induced paranoia, cocaine-linked major depressive episodes [30,31]). In the 2010s, the burst in sequencing technologies development allowed the detection and analysis of hundreds of thousands of single nucleotide polymorphisms. The first GWAS on cocaine related traits, found a SNP belonging to FAM53B significantly associated with CD. Briefly, the analysis of the genome-wide data of 5697 subjects of European (European Americans, EA) and African ancestry (African Americans, AA), together with information from the Semi-Structured Assessment for Drug Dependence and Alcoholism (SSADDA) and incorporating additional data from publicly available GWAS (4063 individuals) found a significant association with the SNP rs2629540 in the FAM53B gene, in both the evaluated ancestries. Furthermore, other additional candidate SNPs were found to be associated separately in EA and AA participants, highlighting the importance of population specific surveys [32]. Notably, the study included among the control samples only individuals that had used cocaine but did not develop any dependence. In doing so, the bias derived from the inclusion of individuals that were liable to CD but were never exposed was avoided. However, a subsequent evaluation of the CD associated SNP role in a sample of 1711 Spanish individuals failed to replicate the findings. This discrepancy could be due to the transferability issue or the different number of analysed markers. The gene FAM53B, located on chromosome 10, regulates the Wnt pathway by regulating β-catenin nuclear (CTNN1) localization, thus playing an important role in cell proliferation and migration, and apparently not related to the occurrence and severity of CD [33]. Nevertheless, a recent study on more than 1 million individuals found that the rs2629540 SNP is associated with a higher educational attainment and a higher ability in math. Considering these discoveries, functional analysis, and expression profile of the FAM53B gene both in humans and animal models could shed light on its role on the occurrence of cocaine, or other substances dependence.

In this context, the in vitro evaluation of genome-wide expression changes after exposure to cocaine in SH-SY5Y cells, derived from bone marrow biopsy aimed to unravel genes that may play a role in CD. This approach revealed that the expression of 756 genes is affected when exposed to 5 µM (but not to 1 µM) of cocaine, with an overrepresentation of genes belonging to many different functional domains involved in the regulation of transcription, intracellular transport, chromatin modification and Neurothrophin signaling pathway [34]. Additionally, a case-control analysis of 22 SNPs that aimed to study the relation between genomic polymorphisms and gene expression changes induced by cocaine, suggested that five SNPs in the untranslated region of the NFAT5 gene could be possibly associated with CD. This result is supported by luciferase assay analysis which highlighted a significant decrease in expression of NFAT5 for the allele rs1437134G under cocaine stimulation. The gene NFAT5 belongs to the Rel family and is involved in osmotic stress response and immune system regulation, and therefore might have a role in the pathogenesis of autoimmune and inflammatory diseases [35]. Like the previously mentioned FAM53B, the functional relationships between this gene and CD are not clear and will require further evaluation. As an example, Gelernter et al. combined data from GWAS [32] with those from transcriptomic analysis, with a particular focus on post-mortem brain sample analysis, and identified additional CD associated SNPs belonging to C1QL2, KCTD20 and STK38 genes. Furthermore, leveraging the data from the Genotype-Tissue Expression (GTEx) project and RNAseq from postmortem hippocampal tissues of 16 individuals (eight cocaine users and eight controls) led to the suggestion that the polygenic predispositions to CD involve molecular adaptations in the hippocampus of cocaine users. Interestingly, the weighted gene co-expression network analysis, which shows correlation in gene expression, revealed that KCTD20 is central in a gene-network significantly associated with cocaine use disorders. Although multi-omics approaches for complex conditions such as CD are desirable, they should be conducted on larger sample sizes, reducing false positives and negatives. A different approach has been to combine expression profiles from humans and animal models, to maximise the chances to identify the loci that are directly related to a specific condition. Combining the expression data from nine studies (four human, three mice and two rats), suggested that many genes might have a role in cocaine metabolism [36].

A recent GWAS performed on 9965 individuals, and including environmental information retrieved in a questionnaire, found 24 genome-wide significant SNPs located in 13 loci for which the effect of the dependence was also correlated with environmental factors. Interestingly, 12 out of 13 loci were not significant if the environmental variables were excluded from the analysis, with the strongest signal found only in individuals of African descent for rs114492924 in LINC01411, encoding a long intergenic non-protein-coding RNA [37]. In particular, individuals with the rs114492924*T allele had higher chances of belonging to a specific cluster of participants characterised by heavy use individuals, early age of onset of CD, and longer period of cocaine heavy use, but only if the effect of the change in residence was taken into consideration. In addition, although not replicated, the authors identified three SNPs that were significantly associated with a CD when the mediation of environmental factors was considered. A deletion in the TMEM51 gene, encoding for a membrane protein, was significant when the variable of “non-traditional paternal care by 13 (years old)” was considered. Similarly, rs149843442 and rs10188036 were significant when household tobacco use and change in residence impact were evaluated. Interestingly, the latter polymorphism is located within the TRAK2 gene, which interacts with the GABA_A receptor, which is in turn potentiated by cocaine, causing the inhibition of dopamine neurons. Overall, these data support the general idea that CD is a multifactorial trait where genetic and environmental factors play a crucial role that need to be deeply explored for a full understanding of the phenotype (Table 1).

Despite the high impact of cocaine use and addiction, the lack of GWAS and their combination with expression data is surprising, and prevents us from fully understanding the biological mechanism behind these traits. The development, creation and maintenance of large population-based biobanks aimed at the analysis and the condivision of different organism data is therefore desirable.

## 4. The Genetics of Heroin and Opioids Use

Opioids are one of the most highly addictive drug classes, and their dependence and abuse are associated with high mortality and morbidity, which is a major societal problem. Opioid Dependence (OD) is moderately heritable [38], and the genetic contribution to opioid abuse has been estimated to be up to 60%, higher than for any other drug class [14,39,40,41,42,43,44].

The isolation of genes that encode opioid peptide precursors opened an era of molecular and genetic investigations of OD [45], and numerous genes and SNPs have been reported to be contributors of OD [38,46]. The study of these genes is therefore useful not only for understanding the pathogenesis of OD, but also for preventing its occurrence and relapse (Table 1).

Early linkage studies have suggested that few genes mapping on chromosome 14 (14q) might have been associated with OD in a sample of 305 sibling-pairs from a mixed population in the US [47], while pioneristic GWAS based on a dataset with a few hundred samples and with no replication tentatively pinpointed OD associated markers [48,49,50].

More recently, a sophisticated and elegant three phase study, based on ~6000 individuals, including replication and independent genotype analysis for more than 2500 samples, found interesting associations between OD and genes linked to calcium and potassium pathways. Many SNPs belong to genes involved in potassium pathways, such as KCNG2 (rs62103177), KCNC1 (rs60349741), APBB2 (rs115368721), and PARVA (rs73411566). Furthermore, a pathway analysis for case-control and symptom counts revealed a significant connection with calcium signalling and synaptic long-term potentiation. Taken together, these results suggest that potassium and calcium transport and signalling mechanisms seem to play essential roles in OD risk. Moreover, a different analysis on the same dataset taking account structural variants highlighted the association of two deletions (18q12.3 and Xq28) and one duplication (1q21.3) with OD [51]. These results, combined with the identification of very rare CNVs associated with a relatively large effect suggest that at least part of the variance related to OD susceptibility might be associated with structural variations, and therefore advocating for high quality genomes [51].

Interestingly, estimates of heritability based exclusively on genetic data have suggested that no less than the 45% of variance in OD features, diagnosed using the Diagnostic and Statistical Manual of Mental Disorders IV was linked to common alleles, characterised by a frequency between 1% and 9%.

A GWAS, including two replication datasets, reported a significant association of five different markers belonging to the CNIH3 gene. Moreover, the integration of these markers with neuroimaging approaches revealed that one of the associated SNP (rs10799590) is also associated with neurophenotypes typically linked to psychopathology resilience [52]. A similar meta-analysis of four different cohorts suggested a role of RGMA gene, which encodes for a protein (repulsive guidance molecule A) involved in many aspects of the development of the adult nervous system [53].

In a recent study, a variant (rs9291211) regulating BEND4 and SLC30A9 in the brain has been also associated with many psychological and behavioural traits, such as depression, alcohol consumption, and neuroticism.

Furthermore, the same study has found that the Polygenic Risk Score for risk-taking behavior estimated on more than 400,000+ individuals resulted in a positive association with OD only when unexposed controls were tested as controls, suggesting that the genomic variants associated with dependence are substantially different from those associated with exposure [54].

After a long debate regarding its association with OD, a recent GWAS on opioid use disorder validates the rs1799971 SNP in OPRM1 gene as associated with the trait. The study involved a greater number of individuals than the previous ones with a sample size of ~100.000 (79.729 EA and 30.061 AA) individuals, finding association only for Europeans [55]. This SNP is a missense polymorphism causing the Asn40Asp substitution, but its biological role in OD is still to be clarified. The same SNP has been studied for the treatment of alcohol dependance with naltrexone, an opioid antagonist targeting particularly μ-receptors. The aminoacidic substitution caused by the missense polymorphism A118G shows an increased response to naltrexone in alcoholics having at least one copy of this SNP [56].

## 5. The Genetics of the Treatment of Substance Use Disorders: A Pharmacogenetics Approach

The findings of genetic polymorphisms possibly associated with several aspects of substance use raised the possibility that at least some of them may also have a role in the level of effectiveness or grade of response to commonly used treatment methods. In the last two decades, many studies have evaluated the correlation between genetic mutations and drug abuse treatment, with a focus on Methadone Maintenance Treatment (MMT) and opioid addiction treatments. Methadone is a chiral molecule, of which the R-enantiomer is characterised by a higher affinity for opioid receptors than its S-counterpart. For this reason, the former is responsible for the therapeutic effect of methadone, while the latter is usually related to adverse effects [57,58,59]. Despite the demonstrated therapeutic value of MMT, its efficacy is also characterised by high variability, both of environmental and biological nature. For example, the pharmacokinetics of methadone show substantial differences among treated individuals, and a study performed on candidate genes suggested that at least part of this variability might be explained by different alleles of the CYP3A4 gene, encoding for the intestinal cytochrome P450. Proteins from the P450 members are involved in the metabolism of drugs and in the synthesis of different lipids, such as cholesterol and steroids. In addition, although measurements of the plasma levels of methadone have been suggested to be harnessed to find the right dose of treatment, a consensus has not been reached so far, and a substantial proportion of patients respond to the treatment only to a limited extent. For this reason, the existence of a link between genetics and response to treatment with methadone has been suggested.

In this context, one of the most investigated SNPs was rs1045642, in the ABCB1 gene (adenosine triphosphate [ATP]-binding cassette subfamily B member 1). However, a recent meta-analysis evaluating seven out of 182 published studies did not confirmed the role of rs1045642 and methadone R or S enantiomers concentrations in plasma, methadone dose and methadone response [60]. On the other hand, the same meta-analysis has shown that individuals with the haplotype CYP2B6*6 were characterised by higher concentrations of R and S methadone.

Thus, additional studies, possibly taking into consideration the whole genomic and environmental background are required to clarify the potential role of this or other polymorphisms.

A recent survey analysing genome-wide data of more than a thousand individuals of European descent did not find any genome-wide significant SNPs associated with methadone dose. On the other hand, for the African American sample, characterised by a lower sample size, the minor allele of the SNP rs73568641, was significantly associated with higher methadone dose. Rs73568641 is located in proximity to the gene OPRM1 (chr6 q24–q25), encoding for the Mu-type opioid receptor (MOR) [61]. The gene, having an important role in pain management, is highly expressed in the central nervous system, in particular in the cerebellum, and when activated by endogenous or analgesic opioids, but also drugs, causes the release of dopamine in ventral striatum and prefrontal cortex. Overall, these results show the importance of population genetics when studying the effect of pharmacological treatments in humans.

A similar survey, analysing 360 Han Chinese patients from Taiwan, and following MMT, have suggested that the SNP rs17180299 can be associated with the plasma concentration of the R-methadone enantiomer, with the minor allele G being characterised by a lower concentration. However, the same SNP was not significant in an independent replication study [62]. On the other hand, a sliding window approach found support for the association between haplotype and different methadone concentration metrics. In detail, four sliding windows (5 SNP long) were associated with blood concentration of R-methadone, with 23 for the S enantiomer. Among the four windows associated with R methadone concentration, three were in chromosome 9, in proximity of and in a LD with the only SNP associated in the canonical GWAS. On the contrary, the windows associated with S-methadone concentration were found close to GSG1L (chromosome 16) and CYP450 genes (chromosome 19). Taken together, the genotype based, and haplotype-based candidate loci explained approximately the 24% and 10% of the S and R methadone concentration, respectively, suggesting that other genetic factors are associated with methadone metabolism. However, it is worth noting that other studies investigating different polymorphisms in the OPRM1 gene, did not find any signal of association [55,63,64].

Another commonly prescribed treatment for opioid use disorder is the combination of buprenorphine and naloxone. Briefly, the former acts as a partial agonist, activating the μ-receptor, and as an antagonist (blocking or reducing the activity) of k-receptor [65,66], while the latter has no agonist properties. A pivotal study has found the SNP rs678849 in the delta-opioid receptor gene associated with both methadone and buprenorphine/naloxone in a sample of 77 African Americans. Interestingly, individuals carrying a homozygous C allele in both chromosomes had a better response to methadone treatment but a poorer one to buprenorphine/naloxone. This result, not replicated on a sample of European Americans, suggests that different treatments might be more appropriate according to the genotype of patients, and could be a key aspect to investigate in future research.

Although the cause of the described discordances in terms of difference in association is currently unknown, and the signals are usually not replicated, this result highlights the necessity of association studies considering multiple different populations and participants from different continental sources, including those commonly underrepresented [67].

## 6. Epigenetics and Addiction: An Overview

Drug abuse causes an increase in dopaminergic stimulation in the nucleus accumbens (NAc), a component of the ventral striatum where the dopamine neurons projecting from the ventral tegumental area terminate [68]. When chronic, drug abuse can lead to long-lasting structural and trascriptional changes by altering the epigenetic signature [69]. These epigenetic changes can be studied on human postmortem brain tissues of drug users [70]. Given the several limitations in performing these analyses on humans, the majority of the current data available have been obtained on animal models, generally rats, exposed to regular and controlled drug doses [70].

Researchers have been mostly focusing on the study of epigenetic changes in cells of the NAc, reporting a global increase in histone acetylation levels, associated with a permissive chromatine state, in response to both acute or chronic exposure to drug [71]. Moreover, in heroin users’ brain, levels of histone H3 acetylation in the striatum correlate with years of use [72], likewise, experimenter-administered or self-administered opioids increase global H3 acetylation within the mesolimbicdopamine system [73,74].

Similarly, chronic cocaine use showed a decrease in H3K9 tri-methylation in NAc, reducing its silencing effect in heterochromatic regions, and a global increase in dnmt3a, regulating de novo methylation, in acute cocaine users. Cocaine also causes a reduction in DNA methylation, while opioids and heroin do not show evidence of this phenomenon [75,76,77].

The presence of these general trends allowed for the identification of the specific sites involved in epigenetic changes. What emerged was a similar effect on both occasional and repeated drug exposure, while drug abuse showed different effects on specific loci according to its duration [78]. Chronic exposure to cocaine and morphine causes an increase in acetylation levels of histone H3 within the promoters of Bdnf and Cdk5 genes, while acute but not chronic cocaine exposure showed increases in acetylation levels of histone H4 of the cFos promoter, as suggested by the lower expression of cFos after chronic exposure [78]. Chronic opioid use also showed lower enrichment of di-methylation of H3K9 throughout FosB gene, increasing its expression as suggested by FosB role as promoter of drug addiction [72].

Other relevant epigenetic modifications are those induced by non-coding RNAs. Although studies are scarce, changes in miRNA activity have been identified after chronic opioid exposure [79,80].

The study of epigenetic changes on DNA offers novel perspectives for the understanding and treatment of drug addiction. In particular, it allows the definition of the neurobiological basis of several phenomena tightly linked to SUDs, such as the maintenance of long-term substance seeking, the hyper-susceptibility to environmental stimuli and triggers (trigger), craving and the relapse even after years of abstinence [81,82]. In this direction, the epigenetics of SUDs explain at biological point of view, the very definition of drug addiction, expressed by the WHO as a “chronic, relapsing disease”.

Additionally, studying epigenetic changes offers therapeutic perspectives for drug addiction: for example, the use of histone deacetylase (HDAC) and histone demethylase (HDM) inhibitors, initially developed for cancer treatments [83]. The need for more studies on this topic remains essential, potentially taking advantage of in vivo neuroepigentic editing approaches. Efforts should be also made to understand the right drug doses for animal models to correctly mirror the human ones and to unravel effects of multiple drugs.

## 7. Conclusions

In this manuscript, we did not intend to provide a systematic review of the extant literature on the genetics of SUDs, but rather a critical overview of the genetics and epigenetics of dependencies, reporting most of the relevant literature with the objective to provide a useful resource for the general public, scientists from different fields and junior researchers.

Our knowledge on the genetic architecture and epigenetics changes underlying traits related to SUDs and their treatments have, after an initial uncertain phase, provided promising results. However, despite these encouraging outcomes, we are still far from a full understanding of such phenomena, limiting a real world application in the management and treatments of SUDs. In particular, from a social point of view, the stability of epigenetics modifications over time and generations, should be deeply studied and considered for its relevant implications for evidence-informed prevention interventions.

In the future, and in an attempt to improve the quality and interpretability of the association studies, many different actions should be taken, namely considering panels of more diverse populations and including evolution-informed features of the variants in the discovery frameworks.

## Figures and Tables

**Table 1:** For each cited gene, further details about the studies reporting their association are given. MMT, Methadone maintenance treatment.

Trait	Gene	Extended Gene Name	Biological Role in Drug Addiction	Sample size (# Individuals)	References
Cannabis use	CADM2	Cell adhesion molecule 2	Unknown	184,765 Europeans	Pasman, J.A., et al., 2018
	SDK1	Sidekick cell adhesion molecule 1	Unknown		
	ZNF704	Zinc finger protein 704	Unknown		
	NCAM1	Neural cell adhesion molecule 1	Unknown		
	NCAM1	Neural cell adhesion molecule 1	Unknown	32,330 Europeans	Stringer, S., et al., 2016
	RABEP2	RAB GTPase binding effector protein 2	Unknown	184,765 Europeans	Pasman, J.A., et al., 2018
	ATP2A1	ATPase sarcoplasmic/endoplasmic reticulum Ca^2+^ transporting 1	Unknown		
	SMG6	SMG6 nonsense mediated mRNA decay factor	Unknown		
Cannabis use (age at onset)	ATP2C2	ATPase secretory pathway Ca^2+^ transporting 2	Possible involvement of calcium signalling mechanism	24,953 individuals	Minică, C.C., et al., 2013
Cannabis use disorder	CHRNA2	Cholinergic receptor nicotinic alpha 2 subunit	Possible direct and indirect interaction of cannabis with the alpha 2 subunit Possible biological link between CHRNA2 and cannabioid receptor genes	2387 cases 48,985 controls	Demontis, D., et al., 2019
Cocaine dependence	FAM53B	Family with sequence similarity 53 member B	Unknown	4498 Europeans 2114 African Americans	Gelernter, J., et al., 2014
	NFAT5	Nuclear factor of activated T cells 5	Unknown	806 cocaine-dependents 817 controls	Fernàndez-Castillo, N., et al., 2015
	C1QL2	Complement C1q Like 2	Unknown	4498 Europeans 2114 African Americans	Gelernter, J., et al., 2014
	KCTD20	Potassium channel tetramerization domain containing 20	Possiblly involved in the distruption of hippocampal gene networks		
	STK38	Serine/threonine kinase 38	Unknown		
Cocaine use disorder	LINC01411	Long intergenic non-protein coding RNA 1411	Unknown	2070 African Americans 1570 European Americans	Sun, J., et al., 2019
	TMEM51	Transmembrane protein 51	Unknown		
	TRAK2	Trafficking kinesin protein 2	Possible interaction with GABA-A receptors when increasing the inhibition of dopamine neuron in response to cocaine		
Opioid sensitivity	KCNG2	Potassium voltage-gated channel modifier subfamily G member 2	Possible involvement of calcium and potassium transport and signalling mechanism	1383 European cases 996 European controls 683 African American cases 2635 African American controls	Gelernter, J., et al., 2013
	KCNC1	Potassium voltage-gated channel subfamily C member 1			
	APBB2	Amyloid beta precursor protein binding family B member 2			
Opioid use disorder	OPRM1	Opioid receptor mu 1	Unknown	10,544 European cases 72,163 Europeans controls 32,088 African Americans	Zhou, H., et al., 2020
Drug use measurement	PARVA	Parvin alpha	Possible involvement of calcium and potassium transport and signalling mechanism	175 Netherlands cases 6268 Netherlands controls	Noordam, R., et al., 2015
Opioid dependence	CNIH3	Cornichon family AMPA receptor auxiliary protein 3	Unknown	1167 Europeans cases 161 European controls	Nelson, E.C., et al., 2015
	RGMA	Repulsive guidance molecule BMP co-receptor A	Unknown	3058 opioid-exposed European Americans	Cheng, Z., et al., 2018
Opioid exposure	BEND4	BEN domain containing 4	Unknown	1297 African cases 2876 European cases 7063 African controls 25,437 European controls	Polimanti, R., et al., 2020
	SLC30A9	Solute carrier family 30 member 9	Unknown		
MMT	CYP3A4	Cytochrome P450 family 3 subfamily A member 4	Unknown	366 Han Chinese in MMT	Chen, C.H., et al., 2011
Methadone Metabolism, Dose and Treatment Response	ABCB1	ATP binding cassette subfamily B member 1	Unknown	1052 opioid dependents	Dennis, B.B., et al., 2014
	CYP2B6	Cytochrome P450 family 2 subfamily B member 6	Unknown		
Therapeutic methadone dose	OPRM1	Opioid receptor mu 1	Unknown	383 African-American Opioid Dependents 1027 European-Americans Opioid Dependent 241 opioid-naive African American children	Smith, A.H., et al., 2017
MMT	GSG1L	GSG1 like	Unknown	360 MMT patients	Yang, H.C., et al., 2016
	CYP450	CYP450 genes	Unknown		
	OPRD1	Opioid receptor delta 1	Unknown	70 African Americans Cocaine and opioid codependent	Thomas, P.S., et al., 2021

## Data Availability

Not applicable.
